# Model of Hydraulic Resistance When Forecasting Reverse Osmosis in Water Treatment

**DOI:** 10.3390/membranes11050314

**Published:** 2021-04-25

**Authors:** Janina Piekutin, Urszula Kotowska

**Affiliations:** 1 Department of Technology in Environmental Engineering, Faculty of Civil Engineering and Environmental Sciences, Bialystok University of Technology, 45A Wiejska Street, 15-351 Bialystok, Poland; 2 Department of Analytical and Inorganic Chemistry, Faculty of Chemistry, University of Bialystok, Ciołkowskiego 1K Street, 15-245 Bialystok, Poland; ukrajew@uwb.edu.pl

**Keywords:** water, reverse osmosis, model of hydraulic filtration resistance, performance prediction

## Abstract

The article presents research on the treatment of infiltration water with increased ammonium ion and nitrate(V) content through reverse osmosis. Then, research was conducted on the phenomena related to the decrease in the permeability of the membrane used for the research. The search for an appropriate interpretation of the phenomena was carried out using mathematical modeling. Based on the assumptions of the hydraulic model of the filtration resistance, calculations were made to forecast the efficiency of the osmotic membrane used in the discussed process. For this purpose, the following indicators were determined experimentally for the membrane: change in the volumetric flow of treated wastewater during low-pressure filtration, total hydraulic resistance, and component resistances, i.e., the resistance of the “new” membrane and resistances resulting from the reversible and irreversible fouling phenomena. It has been observed that irreversible resistance arises in the short and early stages of the process. The efficiency is determined by reversible resistance, which is confirmed by the literature.

## 1. Introduction

The contamination of infiltration water with nitrogen compounds is more and more frequent, which means that classic systems for its treatment are more and more developed. Properly functioning infiltration should remove nitrogen compounds or significantly reduce their concentrations [1,2,3]. The cleaning processes taking place in the soil do not, however, guarantee satisfactory water quality. It is characterized by low turbidity and reduced content of organic substances. The degree of mineralization is much lower compared to groundwater. Due to the possibility of treating infiltration water with increased content of nitrogen compounds using reverse osmosis (RO), there is a need for technological research to learn about all the technical aspects of using the reverse osmosis method for purifying infiltration water [1,2,4,5]. An inherent element of membrane processes is reducing their efficiency related to the accumulation of organic or inorganic substances on the membrane surface, called fouling. This phenomenon occurs mainly in the case of porous membranes, i.e., microfiltration and ultrafiltration membranes, but may also occur in nanofiltration, reverse osmosis, or electrodialysis processes [1,5]. Fouling may be irreversible or reversible [1,2]. Characteristic for reversible fouling is the partial recovery of the membrane permeability due to its cleaning (mainly periodic hydraulic backwash). Particles that are not removed by mechanical or chemical cleaning of the membrane are responsible for the irreversible fouling, leading to the deterioration of the membrane performance. These are mainly particles adsorbed in the pores of the membrane [2,3]. Fouling can be described in terms of total resistance, which includes both the resistance of the membrane and the substances accumulated on the surface of the membrane and in its pores. Fouling also increases the transmembrane pressure during the process under steady flux conditions and a decrease in the permeate flux under constant transmembrane pressure conditions [4,5,6]. The pursuit of maximum efficiency is associated with finding the cause of lowering the permeate flux in the membrane filtration process. The nature of mass transport towards the membrane and back diffusion along the membrane surface (underflow conditions) determines the degree of mass accumulation near the membrane. It makes it possible to determine the resistance to the permeate flux where a precipitate or gel layer has formed on the membrane surface.

It makes it possible to determine the resistance to the permeate flux where a precipitate or gel layer has formed on the membrane surface. Mass transport processes can also resolve the critical conditions for the fouling of solutes on the membrane [1,6,7]. They contribute to lowering the efficiency of the process, thus harming its economy. To ensure the correct operation of the reverse osmosis installation, the rate and degree of blocking of the membranes with the feed should be determined. Membrane mass transport is complicated to model mathematically due to the complexity of the membrane transport mechanism and numerous interactions between all components of the so-called cross effect [6,7,8].

The search for an appropriate interpretation of the phenomena was carried out using mathematical modeling, which will allow researchers to determine the working conditions and learn about the advantages and limitations of using a given method [8,9]. Many mathematical models describe decreasing the volumetric permeate flux during the process of pressure membrane filtration conducted under steady and transient operating conditions. The obtained test results made it possible to verify the model of hydraulic filtration resistances based on the measurements of the change in the size of the RO permeate flux over time, the graphically determined value of the time constant characterizing the reduction of the process efficiency (to a value below economic profitability) and on the experimentally determined value of the resistances: the total membrane, the “not working” membrane, as well as resistances related to fouling phenomena.

### Forecasting the Efficiency of Membranes in Reverse Osmosis in the Process of Purification of Infiltration Water with Increased Concentration of Nitrogen Compounds, Based on the Model of the Hydraulic Filtration Resistance

The modeling of the membrane performance was based on the analysis of the resistance of the flowing liquid membrane and the phenomena caused by the interaction: membrane—substances found in natural waters. The change in resistance in individual test cycles is associated with blocking membranes (fouling). The model of series resistances is based on the equation describing the dependence of the permeate flux on the transmembrane pressure, considering hydraulic resistance of the membrane to the liquid flowing through the membrane, resistance related to reversible fouling, and resistance related to irreversible fouling [7,10,11,12,13,14].

(1) $$J_{v}\;=\;\frac{\Delta P}{\eta \left(R_{m}+R_{fo}+R_{fn}\right)}$$

where:

***J_v_***—temporary volumetric flux of permeate [m^3^ m^−2^·s^−1^];

***R_m_***—membrane resistance value of the ”new” membrane [m^−1^];

***R_fo_***—resistance to reversible fouling [m^−1^];

***R_fn_***—resistance to irreversible fouling [m^−1^];

**Δ*P***—transmembrane pressure [Pa];

***η***—dynamic viscosity of liquid [Pa·s].

The individual components of the resistance were determined:

From the relationship ***J_vH_*_2*O*_** = f(t) for a new membrane with deionized water, the value of the resistance of the new membrane (***R_m_***) was determined. Since under these conditions of the process ***R_total_*** = ***R_m_***, after transforming the Equation (1), we obtain the relationship:

(2) Rm=ΔPη·JH2O

where: ***J_H_*_2*O*_**—experimental temporary flux of deionized water [m^3^ m^−2^ s^−1^],

***η***—dynamic viscosity of liquid [Pa·s].

From a similar relationship, it is possible to calculate the total resistance for natural waters:

(3) $$total=\Delta P/\eta \cdot J_{H2O}$$

where: ***R_total_***—total hydraulic resistance of working membrane [m^−1^] (***R_total_*** = ***R_m_*** + ***R_fo_*** + ***R_fn_***)

***η***—dynamic viscosity of water at 20 °C

The resistance generated by the irreversible fouling effect results from permanent blockage of the membrane, making it difficult to restore its original performance [6,13]. To determine its value, the volumetric flow of the deionized water was determined for a polyamide membrane after reverse osmosis for infiltration water:

(4) $$R_{fn}(\Delta P/\eta \cdot J_{H2O)})-R_{m}$$

where: ***R_fn_***—resistance related to irreversible fouling [m^−1^],

***J_vH_*_2*O*_**—experimental temporary flux of deionized water after pressure filtration plant RO, [m^3^ m^−2^·s^−1^]

By subtracting the corresponding resistance values obtained in the three series of measurements, the reversible resistance ***R_fo_***_,_ and the irreversible resistance, ***R_fn_*** were calculated.

To determine the value of the experimental reversible fouling resistance of polyamide membranes, the following formula (Equation (5)) should be used:

***R_fo exper_* = *R_total_* − *R_m_* − *R_fn_*** (5)

where: ***R_fo exper_***—experimental resistance of reversible fouling.

It was assumed that the changes in the reversible resistance over time could be described by the equation analogous to the Equation (6) defining the changes in the permeate flux over time:

(6) ddt(J0−J∞)+1t0·(J0−J∞ )=0

(7) ddt(R∞−Rfo)+1tRO·(R∞−Rfo )=0

Integrating this equation we get:

(8) Rfo=R∞[1−exp(−ttRO)]

where: ***R_fo_***—initial resistance of reversible fouling (***R_fo_*** = 0 at ***t*** = 0), [m^−1^];

***R_∞_*** resistance of a reversible fouling over an infinitely long period of time [m^−1^];

***t_RO_***—time constant [min].

Equation (9) was used to determine the time constant ***t_RO_***. The value of ***t_RO_*** is determined graphically from the equation:

(9) $$t_{RO}=\left|\frac{1}{a}\right|$$

where: ***a***—the slope of the line described by the equation ***y*** = ***a t***, which characterizes the filtration process for the tested membrane.

After taking the logarithm, the equation of a straight line that passes through the origin of the system of coordinates was obtained. A coefficient ***t_R0_***, which allowed the calculation of the theoretical reversible fouling resistance, was determined from the straight line inclination.

The use of the modeling of the membrane capacity based on the analysis of the hydraulic resistance of the water flowing through the membrane made it possible to determine the values of the individual components of the membrane resistance [13,14]. It was found that the irreversible resistance arising at the very beginning of the process for a membrane operating on the water is lower relating to water with ammonium ion and nitrates(V). It was at the level of 2.300–3.104 × 10^14^ m^−1^ in the infiltration water alone, from 2.272 × 10^14^ to 3.109 × 10^14^ m^−1^ with nitrates(V) and 2.645–3.524 × 10^14^ m^−1^ with ammonium ion.

## 2. Materials and Methods

### 2.1. Subject of Study

The research was carried out using infiltration water taken from infiltration wells. It was carried out in several stages, differing in terms of the adopted technological parameters and the types of infiltration and model water samples (infiltration and enriched with ammonium and nitrates(V)). The treatment efficiency of the tested water was assessed based on the retention coefficient. The following values of pollution indicators were investigated: color, turbidity, conductivity, reaction, calcium, magnesium, total iron, manganese, chlorides, ammonium, nitrates (III and V), and chemical oxygen demand (COD_Mn_). The determinations were made using the test method on a HACH DR 4000 spectrophotometer (Hach Lange GmbH, Düsseldorf, Germany).

### 2.2. Technological Research

In the conducted research, the RO process was carried out in a continuous system with partial recirculation of the concentrate at a small reverse osmosis (RO) station. The tests were carried out on a composite membrane made of aromatic polyamide by Dow Filmtec (Edina, MN, USA) with a spiral module and an area of 2.5 m^2^ in a cross-flow filtration system. The osmotic membrane in the initial filtration was conditioned by filtering the distilled water in the range of transmembrane pressures of 1.1–1.2 MPa to obtain stable operating parameters. The transmembrane pressure was chosen based on the characteristics of the membrane manufacturer. Then, the infiltration water and the infiltration water with added nitrates(V) and ammonium ions were filtered. After 6 h of filtration of the test water through the membrane, the final testing of the membrane with deionized water was carried out for this part of the tests in the same way as in the initial filtration. The above work cycle was considered as one series. To ensure the RO membrane module’s correct operation, the SDI_15_ clogging index was determined for the infiltration water [11]. During the entire research period, the index was determined three times and ranged from 1.29 to 1.7.

## 3. Results and Discussion

During the purification of the infiltration water and model water (infiltration water + nitrogen compounds) performed with the use of RO (Table 1 and Table 2), it was noticed that the retention rates of the chemical compounds oscillated with a slight spread from the mean ± 10%. The turbidity and bacteria were eliminated.

The remaining parameters examined were removed in the range from 71% to 100%, except for nitrates(III), for which the retention coefficient was at the level of from 49% to 51% (Table 2).

**Figure 1:**
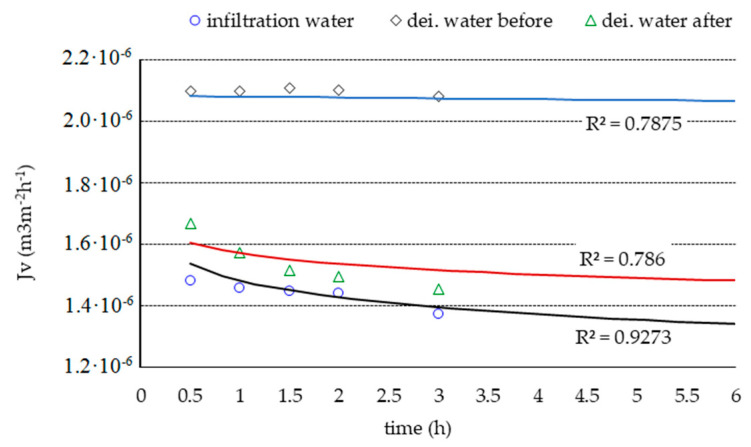
Dependence of the volumetric stream of deionized water and infiltration water on time, where: dei. water—deionized water.

During the purification of the infiltration water (Figure 1), a decrease in the volume flow of water (***J_v_***) was observed in the first half an hour, which then reached the “pseudoconstant” value. A significant reduction in the deionized water stream was observed after testing on natural water. The performed disinfection and rinsing improved the properties of the membranes, although the obtained results were lower than in the test with deionized water performed for the new membrane. The ***J_v_*** value for infiltration water was lower than for deionized water before and after the process (Figure 1).

**Figure 2:**
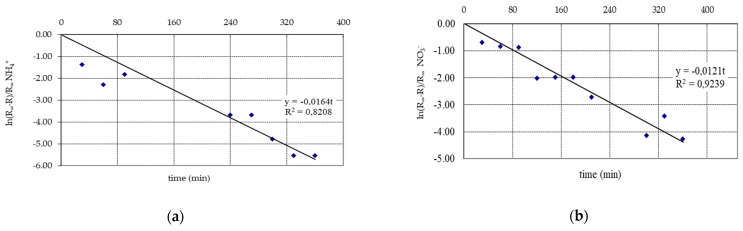
The method of determining the constant ***t_R_*_0_** of the reverse osmosis process: (**a**) NH_4_^+^ enriched infiltration water, (**b**) NO_3_^−^ enriched infiltration water.

To verify the proposed model for the experimental results, the resistance value due to reversible fouling was calculated using Equation (7) (Table 3, Figure 2). The volumetric permeate flux described by Equation (1) was calculated by substituting the values of membrane resistance and irreversible fouling resistance determined from the experiment—the value of the reversible resistance was calculated from the model Equation (7).

**Figure 3:**
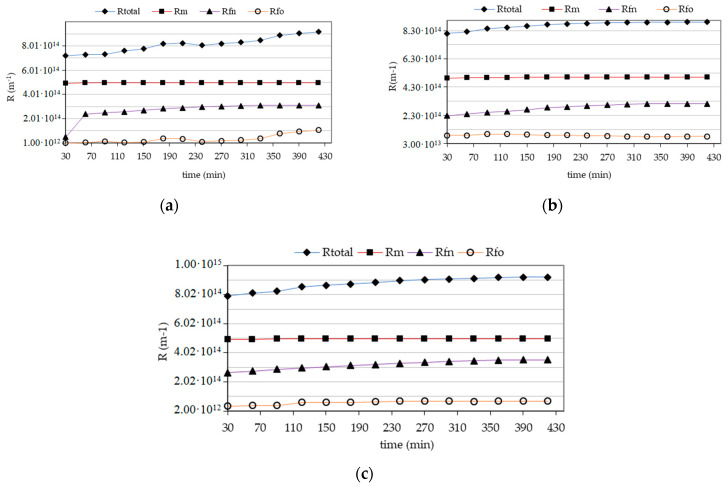
Dependence of changes in resistance ***R_total_***, ***R_m_***, ***R_fo_***, ***R_fn_*** as a function of time for the membrane during filtration of infiltration water and with increased content of NH_4_^+^ and NO_3_^−^, (**a**) infiltration water, (**b**) infiltration water with NH_4_^+^, (**c**) infiltration water with NO_3_^−^.

The obtained dependencies from the model calculations and the experimental measurement points are presented in Figure 1 and Figure 3.

Irreversible resistance in all examined cases systematically increased. However, there were no significant fluctuations or differences between the values of the tested samples. It was also found that in all empirical cases, the total resistance was in the range 7.199–9.17 × 10^14^ m^−1^ (Figure 3). Based on the obtained experimental values of the components of total resistances, the proposed model was verified by calculating the theoretical reversible resistance from the model equation (No. 7), and the coefficient of the equation ***t_Ro_*** determined graphically, and then the theoretical permeate flux was calculated on their basis.

The resistance model of reversible contamination is correct, but requires more experimentation [13,15,16,17,18]. It is necessary to obtain the membrane characteristics with deionized water, both for the new membrane and after the “work” process, to determine the individual components of the resistance occurring in Equation (1) [9,19,20,21,22,23,24].

The most considerable difference between the empirical and theoretical reversible resistance in time ***t_Ro_*** (Table 3) for ***R_fo experim_***_._ 1.829 × 10^13^ m^−1^, and ***R_fo theor._*** 6.974 × 10^13^ m^−1^ was recorded with the infiltration water alone. Much smaller fluctuations occurred in the NH_4_^+^ enriched infiltration water, where the experimental ***R_fo_*** was at the level of 8.722 × 10^13^ m^−1^ and ***R_fo theor_***_._ 5.140 × 10^13^ m^−1^. In the case of NO_3_^−^, the differences were minimal.

The obtained components of total resistance allowed us to determine the dependence of changes in the value of resistance on the concentration of individual nitrogen forms in the infiltration water. Function:

(10) y=a·(1−exp(−b· x))+c

describes the relationship ***R_ck_***, ***R_m_***, ***R_m_*** + ***R_fn_***, ***R_fn_***, ***R_fo_***, ***R_fo theor_***_._ on the concentration of NO_3_^−^, as well as ***R_ck_***, ***R_m_***, ***R_m_*** + ***R_fn_***, ***R_fn_***, ***R_fo theor_***_._ on the amount of NH_4_^+^, while for ***R_fo_*** on the concentration of NH_4_^+^ the function took the form of a polynomial formula:

**y = a + b∙x +c∙x^2^ + d∙x^3^ + e∙x^4^** (11)

There was no significant effect of changes in the concentration of nitrogen species in the infiltration water on the growth of individual resistance values (***R_fo_, R_fo theor._, R_m_***).

**Figure 4:**
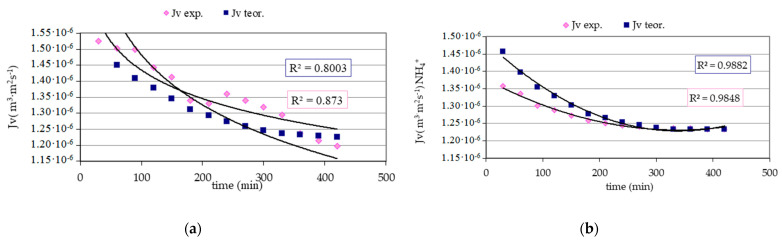

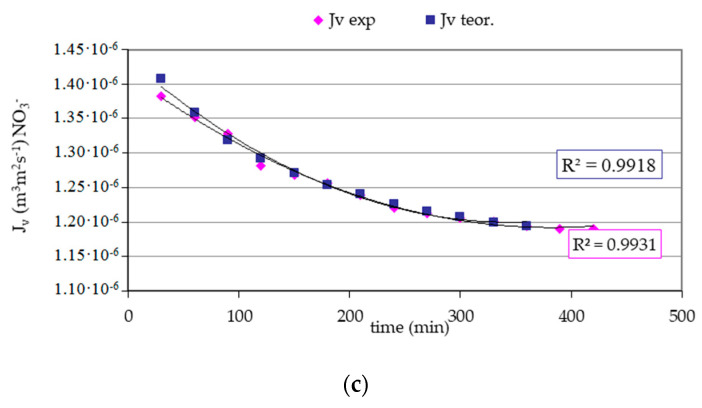
The dependence of the experimental and theoretical volumetric flux (calculated with the reversible resistance model) on time for: (**a**) infiltration water, (**b**) infiltration water with NH_4_^+^, (**c**) infiltration water with NO_3_^−^, where: ***J_v expe_*** − ***J_v_*** experimental, ***J_v theor_*** − ***J_v_*** theoretical.

The obtained values of the empirical and theoretical permeate flux in Figure 4 (calculated with the reversible resistance model) were characterized by slight differences among themselves. In general, the theoretical volumetric flux of permeate achieved results higher than or equal to the experimental one. In both cases, no significant stabilization of the flux was found after some time. For infiltration water, the observed dependence of the volumetric permeate flux on time reached ***J_v_*** = 1.498 × 10^−6^ m^3^ m^−2^ s^−1^ at the beginning of the process, at the end 1.197 × 10^−6^ m^3^ m^−2^ s^−1^, and theoretical ***J_t_*** = 1.525 ÷ 1.225 × 10^−6^ m^3^ m^−2^ s^−1^. During membrane filtration of the water enriched with ammonium ion, the initial value of the theoretical volumetric flux of the permeate was ***J_t_*** = 1.457 × 10^−6^ m^3^ m^−2^ s^−1^, experimental ***J_v_*** = 1.357 × 10^−6^ m^3^ m^−2^ s^−1^, and the final value was identical in both cases and equal to 1.233 × 10^−6^ m^3^ m^−2^ s ^−1^. The relationships between the volumetric flow of the experimental and theoretical filtrate and time for water enriched with nitrates(V) were almost identical and ranged 1.407 ÷ 1.194 × 10^−6^ m^3^ m^−2^ s^−1^. The measure of compliance of the model fit with the experimental values is the calculated values of correlation, which in all tested cases were high and ranged from ***r*** = 0.8730 ÷ 0.9978. Additionally, the calculated percentage of deviation between the volumetric theoretical permeate flux and the experimental one was small and fluctuated from –0.45 ÷ 7.75%.

The verified model of the permeate flux value change based on Darcy’s law [10,14,15,16,17], described as temporal changes in mass transport resistances in the membrane process, was used to identify and estimate the resistances generated in the reverse osmosis process. During the RO process and its components, the total membrane resistance resulting from reversible and irreversible fouling and concentration polarization was determined. It has been shown that irreversible resistance arises in the short and initial period of the process. The efficiency is determined by reversible resistance, confirmed by the literature [18,19,20,21,22,23,24,25,26,27,28,29,30,31]. The values of individual resistances are much higher, relating to the values in other membrane processes. It is related to the more significant pressure difference occurring on both sides of the membrane, which is characteristic of this process [6,9,13,26,27,32]. Based on the research, it was found that the value of irreversible resistance increases over time and is much greater than the reversible resistance. Still, it did not exceed the membrane resistance. It should be assumed that there were substances in the water that tended to form a permanent deposit on the membrane, even though the SDI_15_ index was within the range for reverse osmosis [10,11]. The course of the determined resistances was stable, and the diaphragm’s resistance remained unchanged throughout the research period. The analysis of the obtained resistances relating to water quality confirms the influence of nitrogen forms on their value. The dependence of changes in reversible resistance with time was determined, obtaining a constant presented as ***t_RO_***.

## 4. Conclusions

1. The obtained high values of the correlation coefficients in the case of comparing the instantaneous values of the experimental permeate fluxes with the theoretical instantaneous fluxes allow for the conclusion that that the model of hydraulic filtration resistance used in the calculations allows the forecasting of the membrane efficiency in the discussed process.
2. The resistance model for reversible contamination is correct. In order to determine this resistance, it is essential to obtain the membrane characteristics with deionized water, both for the new membrane and after the “working” process.
3. The value of irreversible fouling resistance is higher than the reversible resistance, indicating additional unrecognized contaminants in the water.
4. The analysis of the experimental data obtained in the process of purification of water with an increased concentration of nitrogen compounds with the use of the series resistances model enables the determination of the primary mass transport resistances, the resistance of the active layer of the membrane, as well as reversible and irreversible fouling, and also the identification and evaluation of the range of phenomena reducing the membrane’s efficiency.

## Figures and Tables

**Table 1:** Effectiveness of removing selected contaminants from infiltration water in the RO process.

Test Parameter	Unit	Raw Water		Infiltrate		Retention Factor R (%)	
		Values		Values		Values	
		Mean	Median	Mean	Median	Mean	Median
Color	mg Pt/L	42.46	44.00	1.23	1.00	97.31	97.42
Turbidity	mg SiO_2_/L	5.64	2.00	0.0	0.0	100.0	100.0
Conductivity	µS/cm	433.0	424.00	7.54	7.00	98.40	98.40
Reaction	pH	7.18	7.20	6.08	6.00	-	-
Calcium	mg Ca^2+^/L	84.93	82.30	0.89	0.00	99.21	100.0
Manganese	mg Mn/L	0.19	0.19	0.05	0.04	77.71	78.90
Ferrum_tot._	mg Fe/L	0.71	0.61	0.03	0.03	95.70	95.71
Chlorides	mg Cl^−^/L	19.30	14.00	0.85	0.70	94.90	93.83
Nitrate(V)	mg NO_3_^−^/L	3.355	3.80	0.045	0.020	99.11	100.00
Nitrate(III)	mg NO_2_^−^/L	0.025	0.025	0.013	0.013	49.60	50.81
Ammonia ion	mg NH_4_^+^/L	0.587	0.45	0.022	0.020	95.10	96.00
COD_Mn_	mg O_2_/L	10.02	9.20	0.43	0.33	89.39	90.15

**Table 2:** The results of the removal of individual indicators at an increased dose of NH_4_^+^ ammonia ion and NO_3_^−^.

Test Parameter	Value	Retention Factor [%]
Color [mg Pt/L]	39.0	74.3
Turbidity [SiO_2_/L]	39.0	100
Conductance [µS/cm]	543	88.45
pH	7.30	-
Calcium [mg Ca_2_^+^/L]	82.6	92.4
Manganese [mg Mn/L]	0.18	75.0
Ferrum [mg Fe/L]	0.67	84.8
Chlorides [mg Cl^−^/L]	16.2	76,0
Nitrate(V) [mg NO_3_^−^/L]	148	71.3
Nitrate(III) [mg NO_2_^−^/L]	0.02	88.0
Ammonia ion [mg NH_4_^+^/L]	12.6	83.6
COD_Mn_ [mg O_2_/L]	10.7	70.7

**Table 3:** ***R_fo_*** resistance values determined from the experiment and theoretical for ***t_RO_***_._

Water Type	Resistance *R_fo_* Determined from the Experiment (m^−1^)	Resistance *R_fo_* Calculated from the Formula (m^−1^)	*t_RO_* Calculated from the Formula (min)
Infiltration water	1.829 × 10^13^	6.974 × 10^13^	156.0
Infiltration water amended with NH_4_^+^	8.722 × 10^13^	5.140 × 10^13^	65.0
Infiltration water amended with NO_3_^−^	4.085 × 10^13^	4.671 × 10^13^	83.0

## Data Availability

Data sharing is not applicable to this article.
